# A digital biomarker of white-matter routing from diffusion MRI as an early biomarker of Alzheimer’s disease

**DOI:** 10.64898/2026.10.21.26366165

**Published:** 2026-10-24

**Authors:** Merit Ghodrat, Johannes CM Schlachetzki, the Alzheimer’s Disease Neuroimaging Initiative

## Abstract

Alzheimer’s disease is diagnosed after a decade-long phase in which pathology accumulates; current markers show that pathology is present, but not how far it has advanced. Because white-matter structure is altered early, we computed two graph-theoretic indices of periventricular white-matter routing from diffusion MRI in 1,706 OASIS-3 sessions: the Route Concordance Index (RCI), a population-calibrated path-concordance score over 316 routes, and a global conductance from the connectome’s graph Laplacian. RCI was reduced before clinical conversion and declined monotonically across a five-stage trajectory (cross-validated AUC = 0.817 for stable-CN vs. pre-AD; comparable to hippocampal volume and exceeding entorhinal thickness, global conductance, and DTI-ALPS), with degradation concentrated in fronto-striatal circuits. A classifier combining these routing features with demographic, structural-MRI and cognitive covariates detected conversion to MCI or AD up to four years before diagnosis (AUC = 0.831; 0.918 within one year; modest positive predictive value). Findings replicated in ADNI ( *n* = 609; conductance AUC = 0.805; RCI = 0.797 under frozen external validation) and tracked CSF amyloid- *β*_42_ in the expected direction. Computed from a standard diffusion MRI sequence, RCI is comparable to established structural and demographic biomarkers on preclinical discrimination while, unlike them, declining cleanly and monotonically across the disease trajectory, supporting prospective validation for risk stratification.

